# Usefulness of diffusion tensor imaging for the differentiation between low-fat angiomyolipoma and clear cell carcinoma of the kidney

**DOI:** 10.1186/s40064-015-1627-x

**Published:** 2016-01-04

**Authors:** Qiang Feng, Zhijun Ma, Sujuan Zhang, Jianlin Wu

**Affiliations:** Department of Radiology, Affiliated Yidu Central Hospital, Weifang Medical University, Weifang, 262500 Shandong People’s Republic of China; Affiliated zhongshan Hospital, Dalian University, No. 6 jiefang Road, Zhongshan District, Dalian, 116001 Liaoning People’s Republic of China

**Keywords:** Diffusion tensor imaging, Renal angiomyolipomas, Clear cell renal cell carcinomas, Fractional anisotropy

## Abstract

To investigate the value of apparent diffusion coefficient (ADC) and fractional anisotropy (FA) in differentiating clear-cell renal cell carcinoma (CCRCC) from low-fat renal angiomyolipomas (RAML), and to obtain the optimal b value. Fifty patients, including 30 cases of CCRCC and 20 cases of low-fat RAML, were retrospectively recruited to participate in this study. Before renal nephrectomy, all subjects underwent functional magnetic resonance imaging. For diffusion tensor imaging (DTI), a respiratory-triggered coronal echo planar imaging sequence was performed with three groups of different b values (0 and 400, 600, and 800). The ADC and FA of kidneys were analyzed and compared between different b values using analysis of variance. Receiver operation characteristic analysis was computed to assess the diagnostic performance of ADC and FA in differentiating low-fat RAML from CCRCC and to determine the optimal b values. With either CCRCC or low-fat RAML, the ADC values decreased with increased b values and significant differences were observed (F = 11.34, 23.15, P < 0.05), while the FA values were not significantly different (F = 0.28, 2.80, P > 0.05). The statistical differences in ADC, and the FA values for CCRCC and low-fat RAML were significantly different (P < 0.05). When the b value was 0.800 s/mm^2^, the cutoff FA value for differentiating CCRCC from low-fat RAML was 0.254 × 10.3 mm^2^/s, and had a sensitivity of 100 %, and a specificity of 73.3 %. MR-DTI can be used to differentiate CCRCC from low-fat RAML.

## Background

Renal angiomyolipoma (RAML) is a benign neoplasm, which is formed by various tissues, including blood vessels, fat, and muscle. Typical RAML can be diagnosed using unenhanced computed tomography (CT), but there is difficulty in distinguishing low-fat RAML from renal cell carcinomas (RCC). However, identification of renal tumor type is essential to the choice of treatment. At present, ultrasound (US), CT, and magnetic resonance imaging (MRI) techniques are inadequate for differentiating benign tumors from malignant renal masses (Paspulati and Bhatt 2006; Kim et al. 2002; Semelka et al. 1991; Israel 2006; Hecht et al. 2004; Ho et al. 2002).

Diffusion weighted imaging (DWI) reflects water motion at the molecular level and provides useful information on parenchymal microstructure and function with the major advantages of not using ionizing radiation or potentially nephrotoxic contrast agents (Notohamiprodjo et al. 2008; Palmucci et al. 2011). The apparent diffusion coefficient (ADC) value is altered by various physiological and pathological conditions of the renal system. Several reports have demonstrated the usefulness of DWI in renal tumor diagnosis (Wang et al. 2010; Kim et al. 2009; Cova et al. 2004; Squillaci et al. 2004a, b; Taouli et al. 2009; Manenti et al. 2008; Zhang et al. 2008; Sandrasegaran et al. 2010; Kilickesmez et al. 2009). DWI is also helpful to characterize and differentiate renal masses and to provide information about the biophysical properties of tissues, such as cell organization and density.

The renal structures of tubules, vessels, and collecting tubules are arranged in a radial pattern, leading to anisotropic distribution (Mannelli et al. 2010). DWI does not allow analysis of diffusion in multiple directions, and therefore diffusion tensor imaging (DTI) is employed. Fractional anisotropy (FA) is a dimensional parameter which analyzes water diffusion in different directions and expresses the preferred direction of the diffusion. When the tissue is completely isotropic, FA = 0, and when the water is forced to diffuse in one particular direction, FA = 1 (Cutajar et al. 2011).

RCC comprises a variety of histological types; therefore, the diffusion property is heterogeneous. Notohamiprodjo et al. (2008) investigated diffusion properties of RCC by DTI, and the surrounding pseudo-capsule of cystic RCC showed a very high FA. The liquefied center showed a very low FA, similar to renal cysts, but with an ADC considerably lower and similar to the solid RCC, so that DTI may have contributed to the differentiation of renal masses. However, there are few comparisons between benign and malignant tumors.

The selection of b value is vital for diffusion imaging. The ADC can be used to evaluate tissue diffusion, which is influenced by both diffusion and perfusion. With low b values, ADC values are greater as a result of tissue capillary perfusion. With a high b value, the effect of perfusion is cancelled out, and the ADC value reflects mostly diffusion. However, higher b values result in signal decay owing to magnetic susceptible artifacts and chemical shift artifacts. Feng et al. (2014) demonstrated that b values (0 and 400, 600, and 800 s/mm^2^) can be used to assess the renal DTI in healthy volunteers. However, the use of different b values to distinguish benign and malignant renal tumors needs further study.

This study investigated the diagnostic efficacy of ADC and FA values in differentiating between clear cell renal cell carcinomas (CCRCC) and low-fat RAML, and determined the optimal b values.

## Methods

### Study samples

A total of 50 cases (30 men and 20 women) admitted to our hospital were randomly recruited retrospectively to participate in this study, including 30 cases with CCRCC and 20 cases with low-fat RAML. The duration of this study was from August 2011 to July 2014. Inclusion criteria are as follows: histologically confirmed renal tumors >15 mm in diameter (3.9 ± 2.5 cm), with unenhanced CT showing low fat; all patients without radiotherapy, chemotherapy, immunotherapy, and biopsy examinations; and, without chronic kidney diseases. Before renal nephrectomy, all subjects underwent functional MRI. The study was approved by the local ethics committee and informed consent was obtained prior to MRI examination.

### MR protocol

All examinations were performed using a 1.5T MR scanner (Avanto, Siemens Medical Solutions, Berlin, Germany) with 32 receiver channels, and a maximum gradient strength of 45 mT/m. For DTI, coronal echo-planar imaging (EPI) sequences were obtained using the six-directional DTI protocol with three groups of different b values (0 and 400, 600, and 800).

The remaining parameters were as follows: repetition time (TR) 1400 ms; echo time (TE), 76 ms; slice thickness, 6 mm with no intersection gap; bandwidth, 1370 Hz/pixel; field-of-view (FOV), 400 mm; FOV in phase direction, 100 %; partial Fourier factor, 6/8; number of excitation (NEX), 4; the k-spaced parallel imaging techniques were generalized auto-calibration partially parallel acquisition (GRAPPA) with an accelerator factor of 2, and acquisition time ranged between 6 min 24 s, and 10 min. The kidneys were consecutively imaged with 10 slices in an oblique coronal orientation.

### MRI data analysis

Two radiologists (WF and ZJM, with 15 and 20 years of experience, respectively) who were blinded to the histopathological results and clinical information reviewed the DTI images. First, the signal intensity of DWI, ADC, and FA maps were evaluated by the radiologists. If there was a disagreement in the judgement, the two observers evaluated the image in a joint session. And then, all measurements were performed at a workstation using Syngo software (Siemens Healthcare, Berlin, Germany).The Neuro 3D Task Card software (Siemens Healthcare) was applied to DTI analyses. The diffusion tensor was determined by the magnitude and the orientations of diffusion. The degree of diffusion anisotropy was analyzed in FA maps, and the ADC were calculated based on a monoexponential fitting model. ADC and FA values were determined separately in all patients. The regions of interest (ROIs) were placed over the solid part of the renal tumor as far as possible, but avoiding the tumor margins. Each lesion was calculated three times and the mean value was adopted. The diameters of ROIs were (29.1 ± 11.9) mm.

### Histology

Formalin-fixed renal tumor tissues were embedded in paraffin and coronal sections (2 μm) were prepared. Sections from routine tissue blocks were examined. Renal histology was analyzed by one pathologist (with 15 years of experience) who was blinded to the results from DTI imaging.

### Statistical analysis

Statistical analysis was performed using SPSS version 13.0 (SPSS, Chicago, IL, USA). P-values of <0.05 were considered statistically significant. The reproducibility between two readers was analyzed by Pearson correlation. Parameters were compared between different b values using one-way analysis of variance (ANOVA) with the least significant difference (LSD) test. Receiver operating characteristic (ROC) analyses were performed to calculate the sensitivity and specificity of ADC and FA according to the threshold which yielded the greatest Youden^’^s index for differentiating low-fat RAML from CCRCC. The cutoff corresponded to the greatest Youden^’^s index value.

## Results

### MR-DTI performance of renal mass

**Fig. 1:**
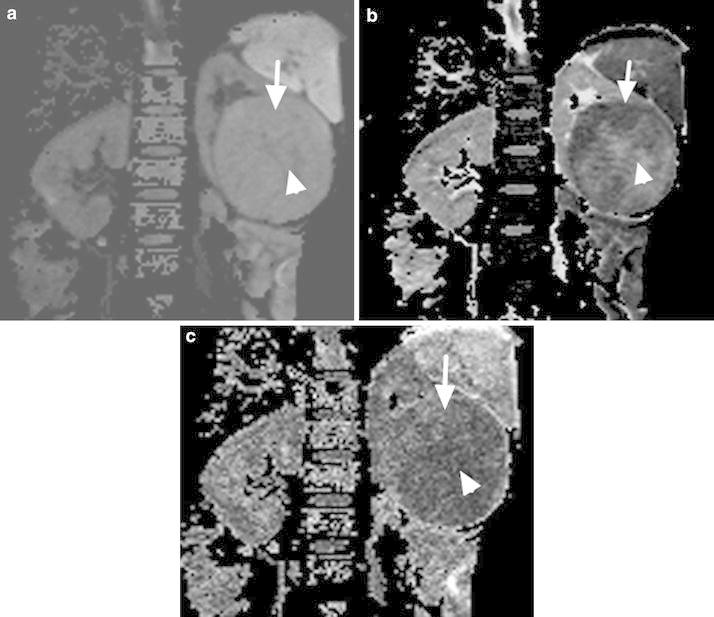
**a**–**c**. Images in 47-year-old man with CCRCC. **a** DWI map the solid parts of CCRCC is higher signal (*arrow*), the necrosis parts show low signal (*arrow head*), **b** ADC map the solid parts of CCRCC is lower signal (*arrow*), the necrosis parts show high signal (*arrow head*). **c** FA map similar to the ADC map

**Fig. 2:**
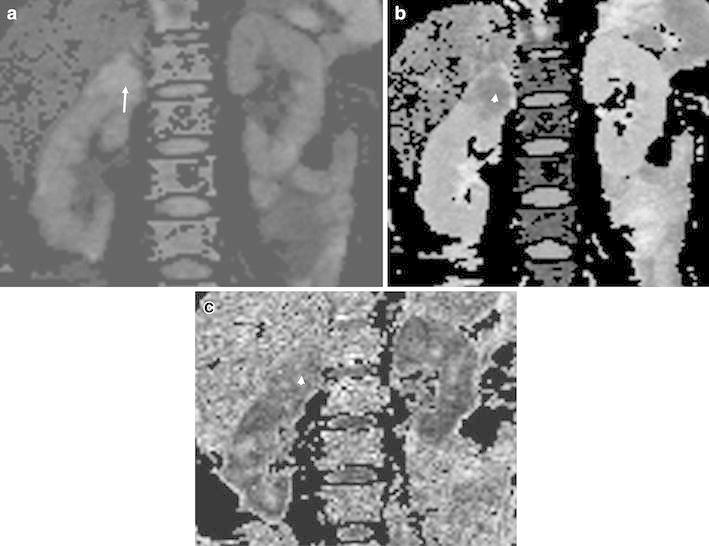
**a**–**c**. Images in 47-year-old woman with low-fat RAML. **a** DWI map RAML show even high signal (*arrow*). **b** Corresponding ADC map shows even low signal (*arrow head*). **c** FA map shows intermediate signal (*arrow head*)

For each patient, the image quality of renal DTI was satisfactory for further evaluation with no motion artifacts, distortion artifacts, or morphological abnormalities. Twenty-five patients with CCRCC displayed necrosis. On the DWI maps, the signals of the solid parts of the CCRCCs were higher than the low-fat RAMLs. The necrosis parts of the CCRCCs showed low signals, while the signals of the solid parts of the CCRCCs were lower than the RAMLs on ADC maps, similar to the FA maps. On the ADC and FA maps, the necrotic parts of the CCRCCs showed high signals (Figs. 1, 2).

### Trend of renal ADC and FA values among different b values

The measurement results of the two readers were close, and the correlation coefficients in ADCs of the CCRCCs, and low-fat RAMLs with three b values were 0.856, 0.832, 0.887, 0.804, 0.846, and 0.901, respectively (P < 0.05), and were similar to the FA values (r = 0.823, 0.863, 0.877, 0.867, 0.903, and 0.891; P < 0.05). The ADC and FA values for three different b values are shown in Table 1. The ADC values in the CCRCCs ranged from 1.12 to 1.51 × 10.3 mm^2^/s. The ADC values in the low-fat RAML group ranged from 1.51 to 1.80 × 10.3 mm^2^/s. Both the CCRCC and low-fat RAML ADC values tended to decrease with increased b value, and significant differences among different b values were observed. However, the CCRCC or low-fat RAML FA values were not statistically significant in the different b values (P > 0.05). The statistical differences of ADC values between the CCRCC and low-fat RAML groups were all significant (P < 0.05), and the FA values were similar.

**Table 1:** The comparison of DTI parameters between CCRCC and low-fat RAML with different b values

b value	CCRCC		RAML	
	ADC	FA	ADC	FA
0.400	1.51 ± 0.36	0.26 ± 0.11	1.80 ± 0.16	0.45 ± 0.15
0.600	1.40 ± 0.34	0.26 ± 0.11	1.75 ± 0.15	0.43 ± 0.10
0.800	1.12 ± 0.26	0.25 ± 0.07	1.51 ± 0.12	0.36 ± 0.07
*F*	11.34	0.28	23.15	2.80
*P*	<0.05	>0.05	<0.05	>0.05

*DTI* diffusion tensor imaging, *CCRCC* clear cell renal cell carcinomas, *RAML* renal angiomyolipomas, *ADC* apparent diffusion coefficient, *FA* fractional anisotropy

### ROC analysis

**Fig. 3:**
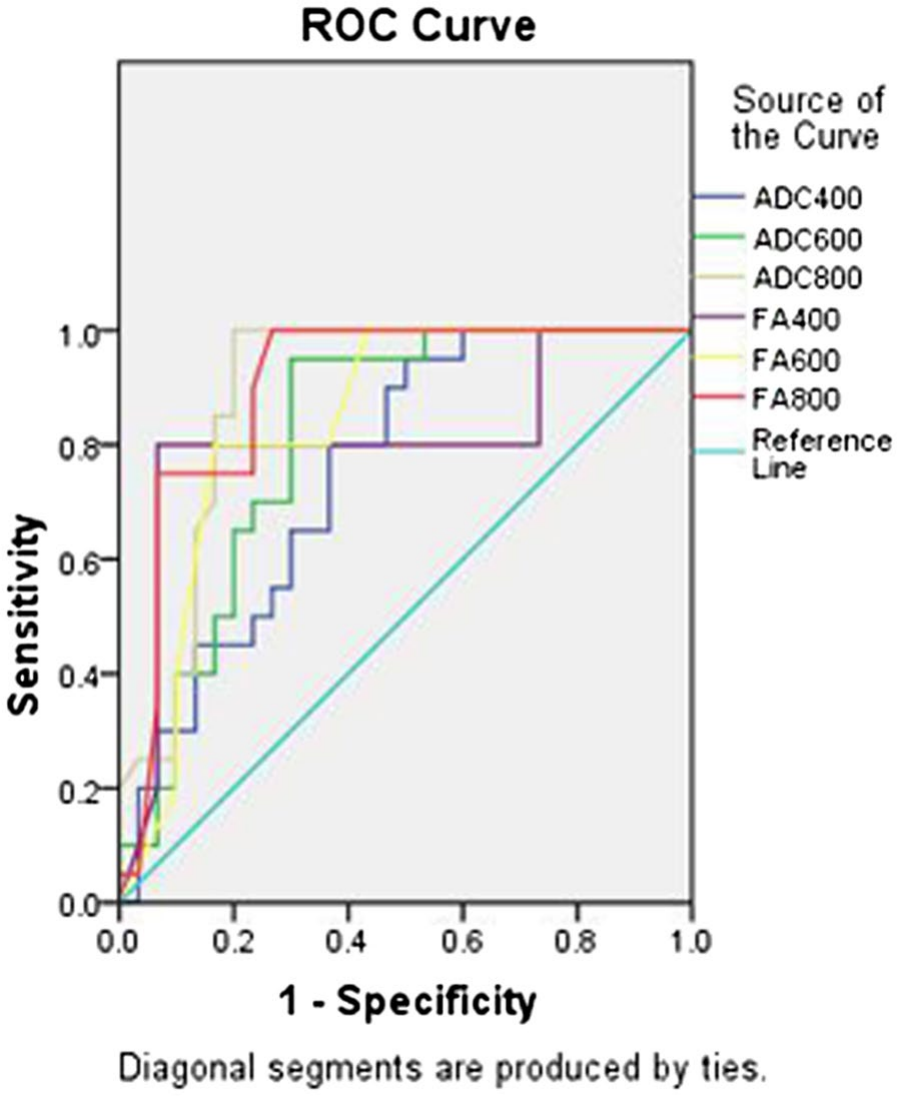
Receiver operating characteristic (ROC) curve of the different parameters and b values show FA value (0.254 × 10^−3^ mm^2^/s) can differentiate CCRCC from RAML with an area under the curve of 0.898. ADC800, FA800 represent ADC, FA value of b (0.800) s/mm^2^

When b values were 400, 600, or 800 s/mm^2^, the areas under the ROC in differentiating CCRCC and low-fat RAML for ADC values were 0.755, 0.813, and 0.888, respectively. The areas under the ROCs for FA values were 0.807, 0.837, and 0.898, respectively. Compared with other b values, the b value at 0–800 s/mm^2^ had an advantage over differentiation between the CCRCC and low-fat RAML groups, and the diagnostic value of FA was the highest. The cutoff for FA value used to differentiate CCRCC from low-fat RAML was 0.254 × 10.3 mm^2^/s with a sensitivity of 100 %, and a specificity of 73.3 % (Fig. 3).

### Pathological analyses of low-fat RAML and CCRCC

The cells of CCRCCs are circular, containing a large amount of lipid, while the low-fat RAMLs are composed of different proportions of smooth muscle, thick-walled blood vessels, and adipose tissue. The smooth muscles of RAMLs were fusiform in arrangement, weaving around the crowed vessels.

## Discussion

To date, several studies investigated the diagnostic performance of DTI in the evaluation of renal diseases. Measurements of DTI parameters within the kidneys, such as ADC and FA, showed low inter- and intraobserver variability in healthy volunteers (Cutajar et al. 2011; Notohamiprodjo et al. 2010). Feng et al. (2015) demonstrated that DTI can also be used to evaluate chronic glomerulonephritis.

In this study, we investigated the values of ADC and FA. The software enabled us to analyze the DTI dataset and view the ADC and FA maps. In this study, a retrospective pattern was designed to investigate the range of these diffusion tensor parameters in benign and malignant renal lesions and to choose the highest efficiency parameters for detecting CCRCC.

Similar to past studies (Taouli et al. 2009; Zhang et al. 2008, 2013; Razek et al. 2011), there is a statistical difference for the ADC value in differentiating renal malignant tumors from low-fat RAML, suggesting that the intensity of tumor cells in solid regions of RCC are higher than that of low-fat RAML. In this study, the difference in FA value between low-fat RAML and CCRCC was significant. The FA values of preceding lesions were both decreased, possibly because the renal space-occupying lesion destroyed the normal renal tissue. The FA value of the low-fat RAML was higher than that of the CCRCC. However, the fusiform structure of the smooth muscles and thick-walled blood vessels in the low-fat RAML and smooth muscle cells surrounded the blood vessels in a radial distribution resulting in increased anisotropy. Residual renal tubules may, however, exist in RAML tissues (John et al. 2006).

Feng et al. (2014) demonstrated that b values (0 and 400, 600, 800 s/mm^2^) can be selected to assess the renal DTI in healthy volunteers. The preceding b values were adopted in the current study, when b values (0 and 800 s/mm^2^) were selected, whether ADC or FA was the highest in diagnosing renal tumor malignancy. A possible reason for this is that the lower b values caused higher ADC values because of the contribution of intravoxel incoherent motion effects other than diffusion (e.g., perfusion). A higher b value provides higher diffusion weighting that is free from perfusion.

The limitations of the current study are as follows: First, we did not include non-CCRCC, such as papillary RCC and homophobic RCC, because their different cellular pathological types may influence diffusion. Second, we did not evaluate the renal tumors <15 mm, therefore, the smaller sample size might have affected the results.

In conclusion, MR DTI demonstrates that b values (0.800 s/mm^2^) gave the highest diagnostic performance. However, MR-DTI may be valuable for differentiating CCRCC from low-fat RAML.
